# The Role of the Inflammasome in Heart Failure

**DOI:** 10.3389/fphys.2021.709703

**Published:** 2021-10-28

**Authors:** Jimin Wu, Erdan Dong, Youyi Zhang, Han Xiao

**Affiliations:** ^1^Department of Cardiology and Institute of Vascular Medicine, Peking University Third Hospital, Beijing, China; ^2^NHC Key Laboratory of Cardiovascular Molecular Biology and Regulatory Peptides, Beijing, China; ^3^Key Laboratory of Molecular Cardiovascular Science, Ministry of Education, Beijing, China; ^4^Beijing Key Laboratory of Cardiovascular Receptors Research, Beijing, China

**Keywords:** inflammasome, heart failure, inflammation, cardiomyocyte, fibroblast, macrophage

## Abstract

Inflammation promotes the development of heart failure (HF). The inflammasome is a multimeric protein complex that plays an essential role in the innate immune response by triggering the cleavage and activation of the proinflammatory cytokines interleukins (IL)-1β and IL-18. Blocking IL-1β with the monoclonal antibody canakinumab reduced hospitalizations and mortality in HF patients, suggesting that the inflammasome is involved in HF pathogenesis. The inflammasome is activated under various pathologic conditions that contribute to the progression of HF, including pressure overload, acute or chronic overactivation of the sympathetic system, myocardial infarction, and diabetic cardiomyopathy. Inflammasome activation is responsible for cardiac hypertrophy, fibrosis, and pyroptosis. Besides inflammatory cells, the inflammasome in other cardiac cells initiates local inflammation through intercellular communication. Some inflammasome inhibitors are currently being investigated in clinical trials in patients with HF. The current evidence suggests that the inflammasome is a critical mediator of cardiac inflammation during HF and a promising therapeutic target. The present review summarizes the recent advances in both basic and clinical research on the role of the inflammasome in HF.

## Introduction

Heart failure (HF) is the end stage of many cardiovascular diseases; its increasing prevalence is a major threat to global health and is associated with enormous economic costs (Benjamin et al., 2019). Inflammation plays an important role in HF, contributing to both its pathogenesis and progression. Proinflammatory cytokines, such as tumor necrosis factor (TNF)-α, interleukin (IL)-1 and IL-6, as well as C-reactive protein (CRP), are upregulated in and related to the severity of HF. Inflammation is also associated with poor outcomes of HF patients and is a prognostic factor that is independent of left ventricular ejection fraction (EF) and New York Heart Association functional class. HF can be categorized as HF with reduced EF (HFrEF; EF < 40%), HF with mid-range EF (HFmEF; EF 40–49%), and HF with preserved EF (HFpEF; EF ≥ 50%), all of which involve inflammation (Castillo et al., 2020; Murphy et al., 2020).

As it plays a major role in inflammation, the inflammasome has been investigated in cardiovascular diseases involving inflammation, for example in HF (Abbate et al., 2020a). The inflammasome is a multiprotein oligomer composed of Nod-like receptors (NLRs), PYD and CARD domain-containing (PYCARD) [also known as apoptosis Speck-like protein containing a CARD (ASC)], and caspase-1, which plays an important role in inflammation and is a focus of cardiac research. Nod-like receptor protein (NLRP)3 inflammasomes are the most widely investigated inflammasomes. NLRP3 is an intracellular pattern recognition receptor that senses pathogen-associated molecular patterns (PAMPs) or damage-associated molecular patterns (DAMPs) and activates the inflammasome and downstream inflammatory cascade. In this manner, the inflammasome is important for defense against bacterial, fungal, and viral pathogens (Tartey and Kanneganti, 2019). However, the inflammasome is also involved in aseptic inflammation. Upon activation, the NLRP3 inflammasome induces the cleavage and activation of IL-1β and IL-18, resulting in inflammation and subsequent cardiovascular injury (Toldo and Abbate, 2018; Zhou et al., 2018; Abbate et al., 2020a). The aim of the present review is to summarize advances in both basic and clinical research on the role of the inflammasome in HF.

## The Pathological Effect of Inflammasome Activation in Heart Failure

During the progression of HF, inflammasome activation by various stimuli promotes cardiac inflammation, resulting in pathologic cardiac remodeling and loss of cardiac cells. Evidence for the role of inflammasomes in hypertrophy, fibrosis, and pyroptosis is presented below.

Nod-like receptor protein (NLRP)3 inflammasome activation has been shown to promote cardiac hypertrophy under pressure overload. Upon this condition, S-nitrosylation of muscle LIM protein (MLP) promoted the formation of a complex composed of toll-like receptor (TLR)3 and receptor-interacting protein kinase 3 (RIP3), which induced the activation of the NLRP3 inflammasome and IL-1β, resulting in the progression of myocardial hypertrophy. Pharmacologic blockade or RNA interference of NLRP3 and inhibition of IL-1β with neutralizing antibody reduced pressure overload-induced myocardial hypertrophy (Tang et al., 2020).

Inflammasomes contribute to cardiac fibrosis under various pathologic conditions. The NLRP3 inflammasome was shown to promote fibrosis progression mainly by stimulating the production of IL-1β and IL-18 (Pinar et al., 2020). The NLRP3 inhibitor MCC950 suppressed myocardial infarction-induced NLRP3 inflammasome activation, alleviated cardiac inflammation and fibrosis, and improved cardiac function (Gao et al., 2019). Chronic β-adrenergic receptor (AR) activation in a pressure overload model and direct acute activation of β-AR led to cardiac fibrosis in an NLRP3 inflammasome-dependent manner (Xiao et al., 2018; Dang et al., 2020). Pressure overload caused activation of the NLRP3 inflammasome via calcium/calmodulin dependent protein kinase (CaMK)IIδ, resulting in cardiac fibrosis and dysfunction (Suetomi et al., 2018). In most of the studies, cardiac inflammation was shown to induce fibrosis following activation of the NLRP3 inflammasome.

In addition to activating IL-1β and IL-18, caspase-1 activated by the NLRP3 inflammasome triggers a type of cell death known as pyroptosis (Miao et al., 2011) by cleaving gasdermin D, yielding the N-terminus of the protein that forms pores in the cell membrane (Zeng et al., 2019). Doxorubicin induced NLRP3 inflammasome activation, triggering cardiomyocyte pyroptosis and thus contributing the myocardial dysfunction and dilated cardiomyopathy (Zeng et al., 2020). Pyroptosis was also shown to amplify inflammation by inducing the massive release of proinflammatory mediators following cell death (Wang Q. et al., 2020). We previously demonstrated that acute activation of β-AR in cardiomyocytes induced NLRP3 inflammasome activation and pyroptosis. Moreover, activated inflammasomes were transferred to neighboring cardiac fibroblasts via membrane nanotubes in response to sympathetic overactivation, leading to amplification of pyroptosis and inflammatory injury (Shen et al., 2020).

Although the pathologic change varies during HF under various pathologic conditions, the inflammasome is shown to play a critical role in the pathologic changes including hypertrophy, fibrosis, and cell death. Thus, it is worth clarifying the mechanism of inflammasome activation during HF.

## Inflammasome Activation in Different Cell Types in Heart Failure

### The Priming and Triggering of Inflammasome Activation

Inflammasome activation is a tightly regulated 2-step process that includes priming and triggering (Latz et al., 2013). Priming involves regulation of the expression of inflammasome components (e.g., NLRP3 and caspase-1) and cytokines (IL-1β and IL-18) by alarmins and DAMPs that activate pattern recognition receptors. This leads to the activation of the nuclear factor (NF)-κB signaling pathway, which promotes the transcription of inflammasome components and cytokines such as NLRP3 and IL-1β (Vallabhapurapu and Karin, 2009). The priming step is necessary for NLRP3 inflammasome activation in the heart. The A350V mutation causes the constitutive activation of NLRP3; tamoxifen-induced conditional expression of the mutant NLRP3 in mouse hearts failed to induce caspase-1 activation, likely because of the relatively low expression level of pro-caspase-1 in this tissue. The caspase-1 was activated in the NLRP3-A350V mutant heart when pro–caspase-1 expression was primed by lipopolysaccharide (LPS) (Toldo et al., 2015), indicating that the priming step is required for active inflammasome formation in the heart.

Triggering refers to the activation of the inflammasome that induces the cleavage and activation of caspase-1 and the subsequent IL-1β and IL-18, leading to inflammation (Dinarello, 2009). As to the most widely studied NLRP3 inflammasomes, triggering involves the generation of reactive oxygen species (ROS), increase in extracellular ATP, cholesterol crystals, and potassium efflux (Heid et al., 2013; Latz et al., 2013; Toldo and Abbate, 2018; Shokoples et al., 2021). The mitochondrial ROS induced the activation of caspase-1 and IL-1β in macrophages following nigericin treatment (Heid et al., 2013). The extracellular ATP released by damaged cells triggered the assembly and activation of NLRP3 inflammasome and subsequent activation of IL-1β and IL-18 through binding to P2X7, a ligand-gated cation channel. Extracellular ATP release and P2X7 activation have been found in many cardiovascular diseases including hypertension, myocardial infarction, and HF (Shokoples et al., 2021). Additionally, cholesterol precipitates as crystals in the vessel wall during atherogenesis. Following the phagocytosis of cholesterol crystals, the lysosomal damage activates NLRP3 inflammasome and IL-1β (Duewell et al., 2010), which was shown to be enhanced by the priming signal of complement (Niyonzima et al., 2020). Moreover, potassium efflux, which causes decreased potassium concentration within cells, is recognized as a common mechanism underlying NLRP3 inflammasome triggering by many NLRP3 activators (such as ATP and nigericin). At low potassium concentrations (< 90 mM), the NLRP3 inflammasome spontaneously assembled and recruited caspase-1 in *in vitro* experiments. Low intracellular potassium level was also required for the NLRP1 inflammasome and may be a common trigger of NLRP inflammasomes (Petrilli et al., 2007; Munoz-Planillo et al., 2013). The detailed mechanism of inflammasome activation has been summarized in recent reviews (Elliott and Sutterwala, 2015; Kelley et al., 2019).

### The Inflammasome in Immune Cells

Immune cells are important in regulating heart functions (Adamo et al., 2020; Sansonetti et al., 2020). The inflammasomes in immune cells contribute to the activation of inflammatory cascade and the development of HF. The NLRP3 inflammasome, caspase-1, and IL-1β are found to be upregulated in peripheral neutrophils from patients with acute myocardial infarction. Calcium-sensing receptor (CaSR) is responsible for inflammasome activation in neutrophils and promotes cardiomyocyte apoptosis and cardiac fibrosis (Ren et al., 2020). Transplantation of bone marrow from *Nlrp3*^–/–^ mice reduced adverse cardiac remodeling following myocardial infarction compared to bone marrow from wild-type mice, suggesting that NLRP3 activation in infiltrated hematopoietic cells increases cardiac remodeling (Louwe et al., 2020).

The accumulation of somatic mutations in hematopoietic stem cells with aging—referred to as clonal hematopoiesis—is an independent risk factor for many cardiovascular diseases (Jaiswal and Ebert, 2019; Sidlow et al., 2020). Mutation of the *Tet2* (Tet methylcytosine dioxygenase 2) gene in hematopoietic stem cells or myeloid cells aggravated cardiac remodeling and function following pressure overload or cardiac ischemia; these effects were inhibited by a selective NLRP3 inflammasome inhibitor MCC950 (Sano et al., 2018). The variations in the *TET2* gene have been linked to an increased level of inflammatory cytokines in patients with severe degenerative aortic valve stenosis or chronic postinfarction HF (Abplanalp et al., 2020). In immune cells of HF patients analyzed by single-cell RNA sequencing, monocytes harboring *DNMT3A* (DNA methyltransferases 3A) gene mutations showed significant upregulation of IL1β and NLRP3, suggesting the involvement of the NLRP3 inflammasome (Abplanalp et al., 2021). Clonal hematopoiesis driven by mutations of *DNMT3A* was found to be associated with poor prognosis in the case of HF (Bazeley et al., 2020). Thus, mutations in myeloid cells induce the activation of the inflammasome in immune cells and the development of HF. Collectively, the inflammasome is critical for the activation of immune cells in inflammatory cascades. Meanwhile, it is also activated in other cardiac cells during HF, contributing to its progression.

### The Inflammasome in Cardiac Cells

Cardiomyocytes have been proved to be a cellular source of secreted proteins, known as cardiokines (Shimano et al., 2012). Under pathologic conditions, cardiomyocytes release inflammatory cytokines (Shimano et al., 2012; Wu et al., 2019). We previously reported that in isolated primary cardiac cells, β-AR agonist activated the NLRP3 inflammasome in cardiomyocytes but not fibroblasts or macrophages, which may be attributable to differential expression of β-AR subtypes across the cell types. Inflammasome activation in cardiomyocytes is mediated by β1-AR, whereas cardiac fibroblasts mainly express β2-AR (Xiao et al., 2018); the activated inflammasome induced the cleavage and activation of IL-18 in cardiomyocytes. The IL-18 stimulated the production of monocyte chemoattractant protein (MCP)-1 by cardiac fibroblasts, which further caused macrophage infiltration, leading to more chemokine production and activation of the inflammatory cascade (Xiao et al., 2018). Inflammasome activation in cardiomyocytes was also observed in other pathologic models. In a mouse model of angiotensin II infusion, CaMKII was shown to mediate inflammasome priming at an earlier timepoint than macrophage infiltration. Cardiomyocyte-specific knockout of CaMKII blocked inflammasome activation (Willeford et al., 2018). A similar result was obtained in a mouse model of transverse aortic constriction: cardiomyocyte-specific knockout of CaMKII blocked inflammasome activation, macrophage accumulation, fibrosis, and cardiac dysfunction (Suetomi et al., 2018). In mouse hearts, inflammasome activation was observed by immunofluorescence mainly in cardiomyocytes 7 days after myocardial infarction. LPS and ATP can prime and trigger the activation of NLRP3 inflammasome in HL-1, the cell-line of cardiomyocytes (Mezzaroma et al., 2011).

The role of inflammasome activation in cardiac remodeling has been extensively investigated in cardiac fibroblasts (Butts et al., 2015). Cardiac fibroblasts are the predominant cell type in the interstitium and can sense cardiac injury and activate the inflammasome, leading to the secretion of inflammatory cytokines (Chen and Frangogiannis, 2013). Priming with LPS and triggering with ATP induced the secretion of IL-1β from cardiac fibroblasts (Torp et al., 2019). In a mouse model of myocardial ischemia/reperfusion injury, the inflammasome was activated in cardiac fibroblasts but not cardiomyocytes. Similarly, in isolated cardiac cells, hypoxia/reoxygenation stimulated the production of ROS and IL-1β in the former but not the latter cell type. The inflammasome in cardiac fibroblasts was found to be activated by ROS and potassium efflux, resulting in the activation of IL-1β and cardiac inflammation (Kawaguchi et al., 2011). NLRP3 also promoted fibroblast differentiation independent of inflammasome formation and was shown to localize to mitochondria and regulates mitochondrial production and Smad signaling, leading to profibrotic gene expression (Bracey et al., 2014).

**FIGURE 1:**
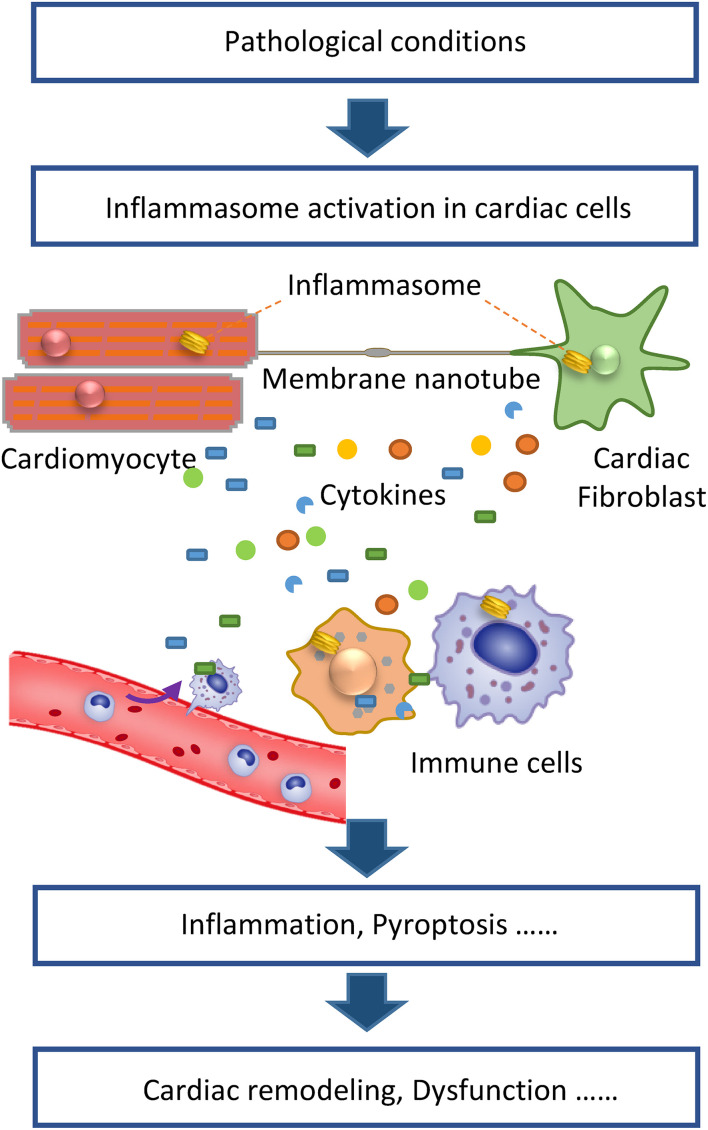
Inflammasome activation in cardiac cells initiates cardiac inflammation to promote heart failure. Under pathologic conditions, inflammasomes in cardiomyocytes, and fibroblasts are activated. Activated inflammasomes cause the release of proinflammatory cytokines from various cell types, collaboratively contributing to the inflammatory cascade. Besides cytokines, membrane nanotubes also mediate the transport of activated inflammasomes, thereby propagating inflammatory injury. The resultant cardiac inflammation causes pathologic cardiac remodeling, cell death, and dysfunction.

The above studies demonstrate that inflammasomes can be activated not only in immune cells but also in cardiomyocytes and cardiac fibroblasts. During HF, these cardiac cells directly sense pathologic stimulation, which induces inflammasome activation and the resultant release of proinflammatory cytokines from these cells. Thereafter, the different cell types interact via paracrine signaling by inflammatory cytokines, resulting in cardiac inflammation. In addition to this indirect mechanism, we have demonstrated that the inflammasome can be directly transferred to neighboring cardiac cells via membrane nanotubes (Shen et al., 2020), a long and thin membrane cellular structure that facilitates the transfer of various molecules and organelles between cells. The exact mechanism of inflammasome transportation in membrane nanotubes is still unknown, whereas the microtubule may be involved as it mediates the transport of other cargos in membrane nanotubes (Shen et al., 2018). Thus, inflammasome activation in cardiac cells may initiate cardiac inflammation under pathological conditions and then different cell types in the heart collaboratively contribute to cardiac remodeling and HF (Figure 1). Moreover, the inflammasome activation in different cell types appears to depend on the conditions. For example, acute sympathetic stress and hypoxia/reoxygenation cause inflammasome activation in cardiomyocytes and cardiac fibroblasts, respectively, via distinct signaling pathways. β1-AR, which mediates the sympathetic stress-induced inflammasome activation, is mainly expressed in cardiomyocytes. Meanwhile, ROS generated under the hypoxia/reoxygenation induce inflammasome activation mainly in cardiac fibroblasts. These findings indicate distinct mechanisms are underlying the role of the inflammsome in HF under different pathologic conditions.

## Inflammasome Activation in Animal Heart Failure Models Under Different Pathologic Conditions

**FIGURE 2:**
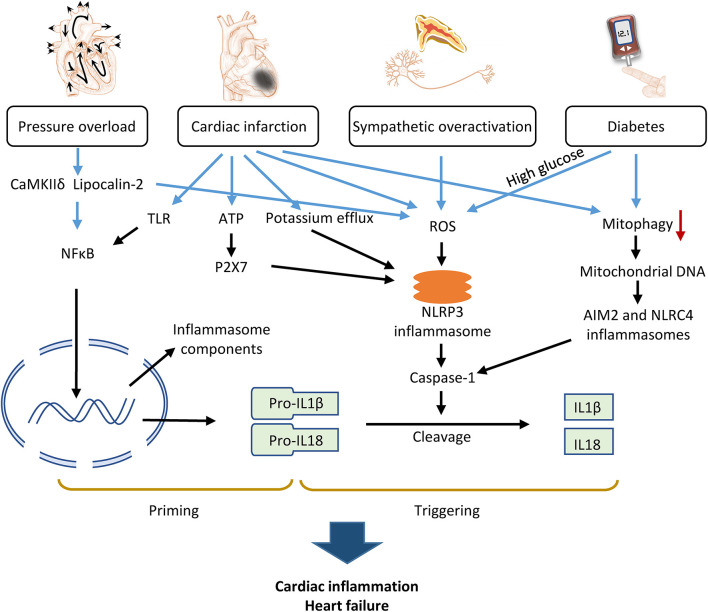
Mechanism of inflammasome activation by pathologic factors contributing to heart failure. Pressure overload, cardiac infarction, diabetes, and sympathetic overactivation caused either priming or triggering of inflammasomes via various molecular signaling pathways, which converge to activate caspase-1 and consequently, IL-1β and IL-18, promoting cardiac inflammation and HF.

Inflammasome activation has been studied using animal models of HF induced by various pathologic factors, such as pressure overload, myocardial ischemia, diabetes mellitus, and sympathetic stress (Figure 2). These basic studies have provided insight into the mechanisms and signaling pathways of the inflammasome in HF.

Pressure overload is a typical cause of HF. Transverse aortic constriction is often used to mimic pressure overload to construct animal models of HF. Heart tissue and isolated cardiomyocytes of mice subjected to transverse aortic constriction showed increased expression of NLRP3 and IL-1β and increased cleavage of caspase-1 and IL-18, indicating that pressure overload increases both priming and triggering of the NLRP3 inflammasome in the heart. The NLRP3 inflammasome activation and subsequent macrophage infiltration, fibrosis, and dysfunction were alleviated by heart-specific knockout of CaMKIIδ and was associated with Nuclear Factor Kappa B (NF-κB) activation and ROS release (Suetomi et al., 2018). These results suggest that pressure overload induces the activation of NF-κB and ROS via CaMKIIδ, resulting in the priming and triggering of the NLRP3 inflammasome and the consequent cardiac remodeling and dysfunction. The role of CaMKIIδ in cardiac fibrosis was also evaluated following angiotensin II infusion, which is another animal model to mimic pressure overload. CaMKIIδ knockout blocked NF-κB activation and inflammasome activation along with macrophage infiltration and cardiac fibrosis in mice treated with angiotensin II (Willeford et al., 2018). Lipocalin-2, a proinflammatory adipokine that was shown to be upregulated in mouse hearts following pressure overload, is another key molecule involved in pressure overload-induced cardiac NLRP3 inflammasome activation. Lipocalin-2 knockout reduced NLRP3 inflammasome activation and alleviated mitochondrial damage. Lipocalin-2 induced the expression of NLRP3 and IL-1β via the TLR4/NF-κB pathway (Song et al., 2017). Collectively, these findings demonstrate that pressure overload promotes both priming and triggering of the NLRP3 inflammasome.

The contributions of the inflammasome and inflammation to atherosclerotic plaque formation and the myocardial infarction have been investigated in multiple studies (Duewell et al., 2010; Xiao et al., 2013; Abbate et al., 2020a) including the Canakinumab Anti-Inflammatory Thrombosis Outcomes Study (CANTOS) (Ridker et al., 2017). Nevertheless, inflammation following myocardial infarction was shown to contribute to the development of cardiac remodeling and HF (Westman et al., 2016). Following myocardial infarction, caspase-1 activity was increased and the inflammasome was formed in heart tissue; NLRP3 silencing or pharmacologic inhibition prevented inflammasome formation and limited infarct size and cardiac enlargement (Mezzaroma et al., 2011). Ischemia during cardiac infarction also contributes to both NLRP3 inflammasome priming and triggering. TLRs are among the major receptors responsible for the priming of the transcription of proinflammatory genes via the NF-κB pathway during acute myocardial infarction; extracellular ATP released from necrotic cells caused the triggering of NLRP3 inflammasomes via P2X7 (Toldo and Abbate, 2018). Triggering is also achieved by increasing the level of ROS via stimulation of thioredoxin-interacting protein (TXNIP) (Liu et al., 2014). Hypoxia/reoxygenation also stimulated inflammasome activation in cardiac fibroblasts through ROS generation and potassium efflux (Kawaguchi et al., 2011). Thus, priming and triggering of the NLRP3 inflammasome contribute to cardiac inflammation and HF development following cardiac infarction.

Diabetes mellitus is a major risk factor for heart disease. NLRP3 inflammasome expression and activation were found to be increased in monocyte-derived macrophages from patients with newly diagnosed type 2 diabetes (Lee et al., 2013). The NLRP3 inflammasome and IL-1β were shown to be involved in the development of insulin resistance and islet beta cell dysfunction (Masters et al., 2010; Vandanmagsar et al., 2011; Jourdan et al., 2013). Intracellular hyperglycemia in diabetes mellitus causes excessive ROS production, which promotes the triggering of the NLRP3 inflammasome (Shah and Brownlee, 2016). NLRP3 inflammasome activation caused cardiac inflammation, cell death, fibrosis, and systolic and diastolic dysfunction in the heart of type 2 diabetic rats, which can be suppressed by NLRP3 gene silencing (Luo et al., 2014). In diabetic cardiomyopathy, Exendin-4 suppressed ROS and TXNIP, resulting in inhibition of the inflammasome and protection against hyperglycemia-induced cardiomyocyte pyroptosis (Wei et al., 2019).

Sympathetic overactivation is the main pathological factor contributing to the development of HF. In our research, we found that the NLRP3 inflammasome plays a critical role in sympathetic stress-induced cardiac inflammation and cardiac injury. Sympathetic stress caused the activation of β-AR, the dominant adrenergic receptor subtype in the heart. Acute β-AR overactivation activated the NLRP3 inflammasome and IL-18 in cardiomyocytes by β1-AR/ROS signaling. The IL-18 then triggered cytokine cascades, macrophage infiltration, and pathologic cardiac remodeling (Xiao et al., 2018). Besides β-AR, α1-AR is also expressed in the heart and we showed that it also contributes to sympathetic stress-induced NLRP3 inflammasome activation in mouse hearts and the resultant cardiac inflammation and dysfunction (Xin et al., 2020). AR-mediated inflammasome activation also occurs in other models of HF. In a rat pressure overload model of HF induced by thoracic aorta constriction, blocking β-AR signaling suppressed inflammasome activation (Dang et al., 2020). A study of HFpEF found that uninephrectomy-induced HFpEF mice exhibited sympathetic hyperactivation and activation of NLRP3 inflammasome and IL-1β (Yang et al., 2020). Growth hormone secretagogue receptor (GHSR) deficiency was shown to aggravate β-AR–mediated cardiac fibrosis in GHSR knockout mice, which involved the increased inflammasome activation and IL-18 cleavage and release (Wang M. et al., 2020). These findings suggest that there is a close link between sympathetic stress and inflammasome activation during the development of HF, although the detailed mechanism remains to be elucidated.

The above studies demonstrate that the inflammasome can be activated by various pathologic factors leading to HF. Although different signaling pathways are activated under pathologic conditions, they appear to converge on inflammasome activation. Thus, the inflammasome may be a common mechanism mediating the development of HF.

## The Role of Other Inflammasomes in Heart Failure

Pattern-recognition receptors other than NLRP3 also form inflammasomes including NLRP1, NLRC4, absent in melanoma (AIM)2 (Table 1); their activation also causes caspase-1 and IL1β/IL-18 activation and is important for the immune defense of infection (Man and Kanneganti, 2015). In a type 2 diabetes mouse model, myocardial infarction reduced left ventricular EF. Mitophagy was impaired in the mice and caused the release of mitochondrial DNA, which activated the AIM2 inflammasome and the NLRC4 inflammasome in cardiomyocytes and macrophages in the peri-infarct region of the left ventricle. Activated inflammasome and caspase-1 caused an increase in cell death and expression of IL-18, resulting in impaired neovascularization and increased fibrosis (Durga et al., 2017; Zhao et al., 2020). AIM2 expression was increased in the heart of streptozotocin-induced diabetic rats, and AIM2 silencing alleviated pyroptosis, cardiac remodeling, and heart dysfunction (Wang et al., 2019; Zhao et al., 2020). AIM2 and NLRC4 expression was also increased in the heart tissue of HF patients and animal models in the late phase of chronic HF induced by pressure- or volume-overload, and following infarction. Activation of the AIM2 inflammasome resulted in activation of both IL-1β and IL-18, and its inhibition with probenecid alleviated chronic HF (Onodi et al., 2021). These findings suggest that AIM2 and NLRC4 are involved in diabetes-related or late-phase HF.

**TABLE 1:** Different types of inflammasomes.

Inflammasome	Activator	Cardiovascular function	References
NLRP1	Anthrax lethal toxin	Myofibroblast differentiation	Zong et al., 2018
NLRP3	PAMPs and DAMPs (bacterial DNA/RNA, virus, ATP, uric acid crystals, etc.)	Hypertensin; diabetes; atherosclerosis; myocardial infarction; cardiac remodeling	Liu et al., 2018
NLRC4	Flagellin, etc.	HF	Durga et al., 2017; Onodi et al., 2021
AIM2	dsDNA	HF	Durga et al., 2017; Onodi et al., 2021
NLRP6	Dextran sodium sulfate-induced colitis	Not investigated in the cardiovascular system	
NLRP12	*Yersinia and Plasmodium infection*	Not investigated in the cardiovascular system	

*AIM2, Absent in melanoma 2; DAMP, damage-associated molecular pattern; dsDNA, double-stranded DNA; HF, heart failure; NLR, Nod-like receptor; PAMP, pathogen-associated molecular pattern.*

In addition to the formation and activation of inflammasomes, AIM2 is known to suppress inflammatory cytokine expression in an inflammasome-independent manner. In mouse cardiomyocytes stimulated with interferon (IFN)-γ and LPS, AIM2 reduced the transcription of proinflammatory cytokines such as IL-6, IFN-γ–induced protein (IP)-10, and TNF-α, by suppressing NF-κB signaling through inhibition of signal transducer and activator of transcription (STAT)1 phosphorylation. The anti-inflammatory effect of AIM2 is independent of caspase-1, as double knockdown of AIM2 and caspase-1 yielded the same proinflammatory cytokine transcriptional profile as observed in AIM2 depleted cells (Furrer et al., 2016). However, this finding is based on *in vitro* experiments and the overall physiologic effect of AIM2 in the heart may be to promote inflammation via induction of inflammasome formation.

## Therapeutic Targeting of Inflammasomes

There is increasing clinical evidence that inflammasomes are activated and involved in HF. In a study of 316 patients with acutely decompensated HF, the concentration of IL-1β, a cytokine activated of inflammasomes, was associated with increased disease severity and risk of death in patients (Everett and Siddiqi, 2019; Pascual-Figal et al., 2019). Increased inflammasome activation was also detected in cardiac biopsy samples from HF patients (Song et al., 2017). A study of 155 HF patients found that the methylation of the inflammasomes component ASC was inversely related to its mRNA and protein expression levels and positively correlated with cardiac function; moreover, elevated expression of ASC was associated with an increased risk of clinical events (Butts et al., 2016). These results provide evidence for the involvement of the inflammasome in the development of HF in patients and indicate the inflammasome as a potential therapeutic target for HF. Some studies have investigated the role of inflammasome inhibition in HF through targeting of either the inflammasome *per se* or its downstream cytokines.

### Preclinical Experiments on Inflammasome Inhibition

A series of NLRP3 inhibitors have been developed that target inflammasome priming and triggering (Mezzaroma et al., 2021). Some of the inhibitors have been evaluated in animal models of HF. Oridonin, the main active ingredient in the traditional Chinese medicinal herb *Rabdosia rubescens*, exerts potent anti-inflammatory activity through specific covalent inhibition of NLRP3 inflammasome. Intraperitoneal injection of oridonin reduced myocardial fibrosis and preserved cardiac function in a mouse myocardial infarction model (Gao et al., 2021). Glyburide, an antihyperglycemic drug, was shown to suppress NLRP3 inflammasome activation by inhibiting ATP-sensitive potassium channels (Lamkanfi et al., 2009). However, there was no obvious protective effect of glyburide against HF (Giles et al., 2010; Siniorakis et al., 2012). Empagliflozin, another antihyperglycemic drug, alleviated cardiac dysfunction in 2 rodent HF models—namely, the HFrEF model, in which mice were subjected to transverse aortic constriction and the HFpEF model, in which rats fed a high salt diet. Empagliflozin attenuated activation of the NLRP3 inflammasome and cardiac inflammation. The mechanism involve restoration of optimal cytoplasmic calcium levels in the heart, as empagliflozin was shown to prevent the LPS-induced increase in calcium level in cardiomyocytes while its anti-inflammation effect was inhibited by the calcium ionophore A23187 (Byrne et al., 2020a).

Some interventions indirectly target inflammasomes. An agonist of REV-ERB—a core driver of circadian mechanism—reduced the expression and activation of NLRP3 following ischemia/reperfusion of mouse heart and thereby prevents the development of HF (Reitz et al., 2019). Chronic elevation of circulating ketones by knockout of succinyl-CoA:3-ketoacid-CoA transferase (SCOT)1 in mouse skeletal muscle suppressed pressure overload-induced cardiac inflammation and cardiac dysfunction. Infusion with β-hydroxybutyrate into isolated hearts inhibited NLRP3 inflammasome activation (Byrne et al., 2020b). Inhibiting of sodium-glucose cotransporter (SGLT)2 increased β-hydroxybutyrate and decreased serum insulin in patients with type 2 diabetes compared to sulfonylurea, and inhibited activation of the NLRP3 inflammasome in isolated macrophages (Kim et al., 2020). Exosomes derived from embryonic stem cells blocked doxorubicin-induced NLRP3 inflammasome activation, pyroptosis, inflammation, hypertrophy, and cardiac dysfunction (Singla et al., 2019).

### Clinical Trials Investigating Inflammasome Inhibition

Therapeutic targeting of the NLRP3 inflammasome or downstream IL-1β signaling in patients with HF has been evaluated in clinical trials (Table 2). Although some of the trials show promising results, most of the studies are still preliminary and future large-scale clinical trials are required for further evaluation.

**TABLE 2:** Clinical Studies targeting NLRP3 inflammasome in patients with HF.

Intervention	Study type	Population	Sample size	Main outcome	References
Allopurinol	Retrospective cohort study	Chronic HF	1760	Increase in mortality (low dose)	Struthers et al., 2002
Allopurinol	Cohort study	HF	6204	Improved survival	Gotsman et al., 2012
Anakinra	Randomized, double-blinded, placebo-controlled pilot study	Acute decompensated HF	30	Well tolerated; reduction in CRP	Van Tassell et al., 2016
Anakinra	Registered clinical trial (NCT01936909)	HFrEF	60	Increase in peak oxygen consumption and ventilatory efficiency at 12 weeks; no effect at 2 weeks	Van Tassell et al., 2017
Anakinra	Registered clinical trial (NCT01950299)	ST-segment elevation myocardial infarction	99	Decrease in incidence of death or new-onset HF; decrease in death and hospitalization for HF	Abbate et al., 2020b
Anakinra	Registered clinical trial (NCT02173548)	HFpEF	31	Decrease in CRP and NT-proBNP; no effect on peak oxygen consumption or ventilatory efficiency at 12 weeks	Van Tassell et al., 2018
Canakinumab	Registered clinical trial (NCT01327846)	Patients with prior myocardial infarction and high-sensitivity CRP ≥ 2 mg/L	10061	Dose-dependent reduction in hospitalization for HF; reduction in composite outcomes of hospitalization for HF or HF-related mortality	Everett et al., 2019
Colchicine	Prospective randomized study	Stable chronic HF	267	Decrease in CRP and IL-6; no effect on primary endpoint rate	Deftereos et al., 2014
Dapansutrile	Phase 1B	Stable HFrEF	30	No serious adverse events; improvements in left ventricular EF	Wohlford et al., 2020
Exercise intervention	Pilot study	HF	54	Increase in ASC methylation; decrease in plasma IL-1β;	Butts et al., 2018

*ASC, apoptosis Speck-like protein containing a CARD; CRP, C-reactive protein; EF, ejection fraction; HF, heart failure; HFpEF, heart failure with preserved ejection fraction; HFrEF, heart failure with reduced ejection fraction; IL-1β/6, interleukin 1β/6; proBNP, brain natriuretic peptide.*

Exploratory results from the CANTOS study showed a significant reduction in the risk of hospitalization for HF or HF-related mortality in patients treated with the IL-1β inhibitor canakinumab (Everett et al., 2019). Anakinra is a recombinant form of the naturally occurring IL-1 receptor IL-1Ra. In a randomized clinical trial of 30 patients with acute decompensated HF, blockade of IL-1 with anakinra significantly reduced CRP level compared to the placebo, suggesting that anakinra suppresses the systemic inflammatory response in HF patients (Van Tassell et al., 2016). Another clinical trial reported that 12-week treatment with anakinra improved peak aerobic exercise capacity in patients with recently decompensated systolic HF (Van Tassell et al., 2017). Anakinra was also found to reduce the incidence of death or new-onset HF and the incidence of death and hospitalization for HF in patients presenting ST-segment elevation myocardial infarction (Abbate et al., 2020b). On the other hand, treatment with anakinra for 12 weeks failed to improve peak aerobic exercise capacity in a group of HFpEF patients with obesity (Van Tassell et al., 2018).

Colchicine is a widely available drug for agouty arthritis and familial Mediterranean fever and can inhibit the NLRP3 inflammasome (Martinez et al., 2018). Colchicine led to a lower risk of ischemic cardiovascular events in patients with recent myocardial infarction and helped to prevent cardiovascular events in patients with coronary artery disease (Tardif et al., 2019; Imazio et al., 2020). However, although colchicine reduced the expression of inflammation-related biomarkers, it did not improve the functional status of patients with stable chronic HF (Deftereos et al., 2014; Hemkens et al., 2016). Allopurinol is another drug to treat gout that inhibits xanthine oxidase, which promotes the formation of oxidative free radicals and induces oxidative stress. Allopurinol suppressed NLRP3 inflammasome activation in cell-based and animal models (Kang et al., 2016; Foresto-Neto et al., 2018; Negi et al., 2020). In clinical trials of HF, allopurinol improved endothelial function and reduced mortality in patients with HF (Struthers et al., 2002; Gotsman et al., 2012; Okafor et al., 2017; Alem, 2018).

A phase 1B clinical trial examining the safety of dapansutrile in patients with stable HFpEF found that dapansutrile treatment for 14 days was well-tolerated (Wohlford et al., 2020; Del et al., 2021). Dapansutrile selectively inhibited the NLRP3 inflammasome but not the NLRC4 or AIM2 inflammasome. It directly targeted inflammasome formation, without affecting potassium efflux or expression of the IL-1β precursor (Marchetti et al., 2018). In a mouse model of ischemia/reperfusion, dapansutrile limited infarct size and alleviated cardiac dysfunction when given within 60 min following reperfusion (Toldo et al., 2019).

Exercise also affects inflammasome activation in HF patients. ASC is required for inflammasome activation, and lower methylation of ASC was shown to be associated with worse outcomes in patients with HF (Butts et al., 2016, 2017). Exercise for 3 months increased ASC methylation and decreased IL-1β and ASC expression following HF (Butts et al., 2018).

## Conclusion and Perspectives

The inflammasome is activated under various pathologic conditions that promote the progression of HF and contributes to cardiac inflammation, making it a promising therapeutic target. The current research on the role of the inflammasome in HF is focused on the NLRP3 inflammasome. The functions of other types of inflammasome in HF—for example, of AIM2 and NLRC4, which were shown to be involved in myocardial infarction injury in a type 2 diabetes mouse model—need to be clarified. The different functions of inflammasomes in HFrEF, HFmEF, and HFpEF also merit further investigation. Inflammation is involved in all forms of HF, but with certain differences (Castillo et al., 2020; Murphy et al., 2020). HFpEF is characterized by the chronic inflammatory state induced by obesity, hypertension, and diabetes. HFpEF mice exhibit hyperacetylated mitochondria, enhanced assembly of the NLRP3 inflammasome, and overproduction of IL-1β and IL-18 (Deng et al., 2021). Meanwhile, HFrEF may be mainly associated with inflammation triggered by DAMPs from direct cardiac insult and cell death. It remains to be determined whether the inflammasome contributes to the different patterns of inflammation observed for HFrEF, HFmEF, and HFpEF. In summary, the current evidence indicates that the activation of inflammasomes in local cardiac cells serves as a trigger for inflammation in HF; clarifying the mechanism underlying this process can yield new insight into the pathogenesis of HF as well as potential therapeutic targets for its prevention and treatment.

## Author Contributions

JW and HX designed the study and wrote the manuscript. ED and YZ revised the manuscript. All authors contributed to the article and approved the submitted version.

## Conflict of Interest

The authors declare that the research was conducted in the absence of any commercial or financial relationships that could be construed as a potential conflict of interest.

## Publisher’s Note

All claims expressed in this article are solely those of the authors and do not necessarily represent those of their affiliated organizations, or those of the publisher, the editors and the reviewers. Any product that may be evaluated in this article, or claim that may be made by its manufacturer, is not guaranteed or endorsed by the publisher.
